# Understanding sustainable cooperation

**DOI:** 10.1007/s11186-025-09647-x

**Published:** 2025-09-30

**Authors:** Rafael Wittek, Bas van Bavel, Naomi Ellemers, Martin van Hees, Tanja van der Lippe, Russell Spears

**Affiliations:** 1 Department of Sociology, University of Groningen Groningen, Groningen, Kingdom of the Netherlands; 2 Department of Economic and Social History, Utrecht University, Utrecht, Kingdom of the Netherlands; 3 Department of Organizational Behavior, Utrecht University, Utrecht, Kingdom of the Netherlands; 4 Department of Moral and Political Philosophy, VU University, Amsterdam, Kingdom of the Netherlands; 5 Department of Sociology, Utrecht University, Utrecht, Kingdom of the Netherlands; 6 Department of Social Psychology, University of Groningen, Groningen, Kingdom of the Netherlands

**Keywords:** Sustainable cooperation, Institutions, Social mechanisms, Micro-macro link, Meso-level

## Abstract

Societies need cooperation that is sustainable. We argue that understanding the mechanisms of sustainable cooperation requires a connection between current analyses of individual interactions and their institutional contexts, and studies of long-term patterns of cooperation at the societal level. We propose a focus on where institutional level arrangements connect with individual level decision making, namely where people interact in families, communities, and organizations, i.e. the “meso-level” of society. Focusing on the impact of external threats, spillover effects and vicious cycles, our transdisciplinary approach highlights the importance of understanding when and how individual and institutional dynamics can undermine cooperation at this intermediate level and what is needed to secure cooperation sustainability in society.

## Introduction

Cooperation, or the joint production of mutual benefits, requires fruitful interactions between individuals. Whether we are looking at market agents, family members, policy makers, or colleagues within a firm, cooperation is essential for the success of their interactions: without it markets do not function, families will break down, firms are unable to achieve their objectives, and political objectives become unattainable. Many efforts to understand cooperation have focused on the question of how to get cooperation going: the *emergence* of cooperation has received a lot of attention and its study now is a well-established area of research.

However, cooperation can also break down. It may no longer yield benefits to its participants or may create unacceptable negative effects for third parties. We need an understanding of the nature of *sustainable* cooperation, namely: insights into the mechanisms that ensure the continuation of cooperation as well as the conditions under which its existence is valuable. We argue that this understanding can be achieved by combining approaches that examine individual and institutional dynamics, respectively, to determine when and how social interactions promote or hinder cooperation.

Solving problems of sustainable cooperation requires closing the gap between micro-level approaches that focus on individual preferences but neglect their contextual embeddedness, and macro-level approaches that capture the intricacies of institutional contexts, but without specifying causal processes at the individual level. The most effective way to connect these two approaches, we suggest, is to focus on the intermediate arrangements that keep cooperation going within and between families, communities and organizations, i.e. the “meso-level” of society (Streeck & Schmitter, 1985). While there is a host of studies on the functioning of families, communities and organizations, and how people interact within them, there is scope for more systematic analysis of how individual and institutional dynamics impact on this intermediate level. It requires examining how micro-level (such as individual priorities, behavioral preferences, and social connections) and macro-level forces (such as institutional structures) affect the sustainability of cooperation in communities, families, organizations (Turner, 2012).

We will argue that a further analysis of conditions that threaten cooperation at the meso-level is key to understanding the sustainability of cooperation and for developing testable predictions about the conditions that undermine and foster it. More specifically, we consider three types of threats to be of particular relevance: external shocks (such as the COVID pandemic) that challenge existing meso-level arrangements, negative spillover between meso-level domains (such as work demands impinging on family arrangements) and vicious cycles (e.g., meso-level arrangements that undermine individual motivation to cooperate).

## Current approaches to cooperation

Applying a large variety of methods and theoretical approaches, the social and behavioural sciences have made enormous progress in understanding the emergence of cooperation as well as some of the conditions under which it can be sustained (Nowak & Sigmund, 2005; Gächter et al., 2017; Wittek & Bekkers, 2015). We discern two insufficiently connected strands in this literature.

First, studies using experimental games, measures of brain activity and computational models have elucidated individual differences, perceptions of interdependence, and coordination rules that help people achieve cooperation. Behavioural studies have improved our insights into how institutional structures affect the behaviour of individuals operating within them. Formal modelling efforts help designing institutional mechanisms that shape new patterns of successful cooperation or that secure and bolster existing ones (Maskin & Sjöström, 2002; Parikh, 2002).

Second, empirical studies of emergence and erosion of cooperation examine the development of institutions over longer periods in time. These studies, often conducted by sociologists, economists and historians, extend the focus from cooperation between individuals to cooperation between groups and societies (North, 1990; Ostrom, 2000; Kuran, 2004; Acemoglu & Robinson, 2012; Williamson, 1985). Such studies may involve formal modelling but more often apply descriptive and explanatory methodologies, including empirical tests of hypotheses derived from theoretical approaches (e.g. through comparative analysis) (Van Bavel, 2015).

Despite these advances, there is a *lack of integration* between the insights and results from studies focusing on individual behaviour and its explanation, and large-scale empirical studies of institutions. Thus, there still is a disconnect between explanations at the micro- (individual) and the macro-level (society) within the study of cooperation. This indicates scope for theoretical advancement by focusing on the meso level, which connects the two.

### Sustainable cooperation

Cooperation is sustainable when it is *stable* as well as *valuable*. The mere continuation of cooperation does not make it sustainable. Even if enduring cooperation can reinforce group solidarity, a negative impact on individual or societal resources can make it unsustainable. This shows that stability over time is not the only criterion that determines the sustainability of cooperation. Indeed, sustainability also depends on the ethics of cooperation, and the nature of the values at stake. Over time, the stability, the value of the cooperation, or both, can decline. We note that individual and institutional dynamics can undermine the sustainability of cooperative arrangements at the meso-level. We identify external shocks, negative spillovers and vicious cycles as relevant threats to the stability and/or value of the cooperation and hence to cooperation sustainability. An examination of these dynamics may provide the desired link between the micro- and macro-analysis of cooperation.

### Stable cooperation

Successfully created cooperation need not be stable. In fact, there is considerable evidence of cooperation breaking down - even after conditions favouring cooperation have been created. Experimental studies of iterated N-person dilemmas, for instance in public good games, consistently find that cooperation decreases significantly over time (Ledyard, 1995; Fehr & Gächter, 2002), beginning to wane almost from the start. This decline is even more dramatic in situations where the explicit aim is to *maintain* the cooperation (e.g. by limiting what one takes from a collective outcome pool), compared to situations where the goal is to *create* a collective good by contributing to it (Gächter et al., 2017).

Behavioural as well as institutional approaches have focused on monitoring and sanctions as a way to curb such lack of cooperation, for instance by identifying and punishing free-riders to safeguard stable cooperation (Fehr & Gächter, 2002). But the cure can be worse than the disease. In fact, the limitations of sanctions as a means of promoting cooperation have been well-documented. In small groups there are social costs to punishment (Guala, 2012), and deterrence sanctions raise mutual distrust that invites lack of compliance with cooperation requests (Mooijman et al., 2017). In general, formal monitoring and negative sanctions decrease the intrinsic motivation to cooperate, causing it to break down as soon as monitoring efforts are weakened or sanctions become more relaxed (Ostrom, 2000). Likewise, at the macro level it has been shown how cooperation in markets in the longer run may transform into antagonistic or coercive relations or lead to inequalities that make people and groups retreat from the market (Van Bavel, 2016).

### Valuable cooperation

To say that cooperation is sustainable is also to say something about its value (Bicchieri, 2005). The value judgement contains two parts. First, the value that cooperation has to those involved: it yields benefits that they could not have obtained on their own. These benefits may be economic, for instance when people cooperate to obtain financial gains. They may consist of new possibilities (improved prospects, new opportunities), for instance when family members support each other in their personal development. They may also refer to certain values that are inherent to the practice itself, as when an organisation attaches importance to diversity and inclusion, or a community to caring for the weak or vulnerable.

A second requirement is the social value of cooperation. A prospering local community not only enhances the well-being of its residents but is also able to contribute to social cohesion more generally, for instance by accommodating newcomers (Sampson, 2003). Similarly, when workers cooperate in a firm with the result that their joint efforts enhance the firm’s profitability, this is only sustainable when it does not unduly burden the natural and social environment. Individual value and social value need not always coincide. Cooperation may be stable and may have value to the people involved without it having much social value. What is crucial in our conceptualization of sustainability is that the cooperation should not have *negative* social value. A criminal organization or a xenophobic community are not sustainable according to this conceptualization, even when they are stable and have value to the individual members.

## Threats to sustainable cooperation

To anticipate and address threats to sustainable cooperation, institutional models should specify the implicit behavioural theory on which they are based (Powell & Colyvas, 2008), instead of assuming that individual preferences can be taken as exogenously given (Manski, 2013; Riedl, 2010) or emerge consistently across cultures and societies (Henrich et al., 2001). Nevertheless, empirical studies of institutional mechanisms that focus on long term sustainability of cooperative relationships, over decades or even centuries, often do so only at the level of whole societies or regions (Kuran, 2004; Acemoglu & Robinson, 2012). This makes it difficult to connect to insights from behavioural research that reveals considerable behavioural variability across institutional settings and help understand sustainability threats. Nevertheless, a consideration of individual as well as institutional dynamics is needed to account for the patterns of cooperative behaviour documented in research.

### Individual dynamics

Institutional explanations of individual behaviours often assume that preferences are exogenously given and fixed (Gerber & Jackson, 1993). In line with a growing literature also in economics (Alger, 2023), we argue this does not do justice to current knowledge about the different sources of individual motivation documented in psychology and sociology, based on situational variations in people’s social identities (Scheepers & Ellemers, 2019), social networks (Schweitzer et al., 2009), and the ways goals are framed (Keizer et al., 2008). When preferences are thus endogenized, they cannot be assumed to be fixed: relevant social identities, networks and goal frames are known to vary over time and across meso-level contexts.

The concept of *social identity* refers to “that part of the self derived from our membership of social groups together with the value, meaning and emotional significance attached to this” (Tajfel, 1978). This highlights the subjective meaning of given categories that can shift over time and across (meso-level) contexts and complements analyses assuming individual motives from category memberships (Turner, 2012). The notion that we can define ourselves at different levels of social inclusiveness suggests we sometimes internalize the interests of other individuals included in the group with which we self-identify. This happens for instance in families, organizations, or communities, when justice concerns or care responsibilities trump self-interest as cooperation motives. Situational shifts in social identity salience also explain cooperation trade-offs between meso-level identities and predict for instance, when workers sacrifice their family time to achieve organizational goals, or refuse to do so. Thus, social identities not only extend self-interested behaviours to the collective level of in-group favouritism. People sometimes even make ‘irrational’ choices that incur personal costs and sacrifice in-group gain when this helps them define their distinct social identity (Spears & Otten, 2025).

Further, even when anticipated gains and interdependence structures remain the same, people shift in their preferences for *whom* to cooperate with. This depends on the development of interpersonal networks - webs of relationships based on repeated interactions with specific others - which make it easier to sustain cooperation with some than with others. Network relations define and reinforce reputations (e.g. about trustworthiness), the creation of mutual obligations (reciprocity), and enable informal monitoring and sanctioning, for instance through gossip or social exclusion (Buskens & Raub, 2002; Ostrom, 2009). Importantly, a growing number of studies demonstrate the dynamic nature of these social networks and how they affect cooperation, since personal networks may change as a result of the behaviour they trigger, and vice versa (Fehl et al., 2011).

Finally, research on *goal framing* clearly demonstrates how dynamics in the social environment can trigger cooperative or non-cooperative behaviour, for instance by making salient gain motives (directed towards the long term improvement of one’s resources), hedonic (i.e. oriented towards short term need satisfaction), and norm adherence (i.e. acting appropriately in a given context, even if this comes at a cost) (Lindenberg & Steg, 2007; Keizer et al., 2008). These goal frames differ in their a-priory strength, with the normative goal frame being the most brittle one. This means that situational cues related to gain or hedonic goals may easily weaken the impact of institutional norms towards (costly) cooperation. Hence, understanding sustainable cooperation requires an analysis of conditions that activate the normative goal frame and keep it in the cognitive foreground (e.g. Wittek et al., 2003).

### Institutional dynamics

By definition institutional arrangements champion a specific solution to facilitate cooperation. Since they are meant to offer stability, they typically do not anticipate or provide for institutional change, which often only emerges in a crisis, for instance as a result of external shocks (Kingston & Caballero, 2009). Recent research also documents the long-term persistence even of inefficient institutions (Eggertsson, 2009; Kuran, 2004), and shows how rarely endogenous institutional change occurs (Aoki, 2007). If an institutional arrangement endogenously generates gradual changes that in the long run undermine the extent to which the institutional system and the associated behaviour are self-enforcing, this is mostly unintended and may lead to the demise of that arrangement (Greif & Laitin, 2004; Herzog et al., 2024).

A dynamic view on the link between institutions and cooperation acknowledges that the kind of arrangements that were successful in bringing cooperation about might not be successful in keeping it going. There is ample evidence of the potentially self-undermining effects of specific and well-known governance practices. These include the perverse incentives that may emanate from performance contingent pay (Prendergast, 1999), or of inconsistent combinations of governance practices (Baron & Kreps, 1999).

Specific institutional arrangements, once adopted, might be difficult to change, despite the availability of more efficient alternatives (Mahoney, 2000). This includes the emergence of institutional sclerosis (Olson, 1984), in which those who benefit from an institutional arrangement have an interest in maintaining the status quo and hence cooperate in blocking reforms that attempt to create more inclusive economic and political institutions (Acemoglu & Robinson, 2012). This can have devastating effects on social welfare and ultimately undermine the institutional arrangements that were set up to enable cooperation between societal stakeholders (Van Bavel, 2016). Institutional arrangements thus need to offer scope for adaptation to changing circumstances, or even transformation, to be able to achieve sustainable cooperation (Bicchieri, 2017; Folke et al., 2010).

## Unpacking the elements of cooperation sustainability generates new predictions

The proposed framework elucidates how micro- and macro-level forces can foster or undermine the sustainability of cooperation at the meso-level of families, communities and organizations. Three types of potential threats to cooperation sustainability need to be distinguished.

*External shocks* refer to events that are rare and difficult to predict, and that have their origin in the macro-level realm - including its institutional, cultural, physical and natural environments - of the meso-level unit under investigation. Examples of such external shocks that form threats, i.e. that may have a negative impact on cooperation, are earthquakes, financial crises, pandemics, or large scale armed conflicts.

*Spillover effects* concern situations in which the dynamics in one meso-level unit affect cooperation in another meso-level unit. Examples of negative spill-over effects are work-life balance issues where growing commitments at the work organization can negatively affect family life.

*Vicious cycles* are self-reinforcing processes - “positive feedback loops” with negative consequences (Masuch, 1985) - that originate within the meso-level unit under investigation and that undermine cooperation within it. An example are dysfunctional family interactions – such as a lack of communication, parental rejection, and frequent conflict – that increase stress and reduce emotional support for family members. This may lead to low self-esteem and problematic behaviors, which in turn further worsens family relationships.

Acknowledging that each of these three processes can negatively affect the stability and value of cooperation not only allows better diagnoses of threats to cooperation sustainability, but also leads to better informed strategies to prevent their future occurrence or mitigate their consequences. The following examples and related propositions illustrate.

### External shocks

When an *external shock* undermines the institutional arrangements that secure stability of cooperation, our analysis implies that individual-level dynamics playing out at the meso-level predict continued willingness to cooperate. An example of how this might work was observed in the behaviour of healthcare interns in The Netherlands during the COVID pandemic (Teekens et al., 2021). The first lockdown disrupted institutional arrangements as interns were allowed to stay at home to prevent them from becoming infected at work. Nevertheless, a substantial proportion of 23% kept going to work, despite the lack of instrumental incentives (school guidelines), the potential costs for their personal health, and the absence of long-standing commitment to their host organizations. In line with our reasoning, the continued attendance was explained by the high level of task interdependence and a collaborative organizational culture at the meso-level. This example suggests that the subjective importance of one’s contribution to the group can compensate for the breakdown of cooperation incentives, and determines the sustainability of individual investment into the continued cooperation.

### Negative spillover

*Negative spillover* effects can shift the value of the cooperation and hence undermine its sustainability. Such value shifts occur when processes in one meso-level context, like work organizations, have negative repercussions for cooperation in another valued context, like families or communities. Indeed, research documents the impact of institutional contexts on individual values and goals (Do Canto et al., 2022), for instance in prioritizing monetary rewards of paid labour or the provision of care (Koster et al., 2021). This analysis sheds new light on the phenomenon that is known as “quiet quitting” (where employees perform only minimum duties listed in their job descriptions), which is often attributed to younger generations attaching more value to their personal well-being and development than to work. Instead of pointing to generational differences, our analysis predicts that continued work engagement is feasible when organizations prevent negative spillover by aligning their mission and practices with community and family values that are important to their workers. Indeed, organizations can counter employee disengagement and reduce quiet quitting by adjusting existing arrangements to address emerging causes of work dissatisfaction, for instance improving organizational support for working parents (Blom et al., 2025; Dillard et al., 2025).

### Vicious cycles

*Vicious cycles* threaten cooperation sustainability because they undermine the stability as well as the value of cooperation. Attempts to enforce cooperation with increasing controls and sanctions typically antagonize people and invite a search for loopholes that reduce voluntary compliance. Our analysis instead encourages institutional decision makers to consider the motivational and behavioural effects of their policies, aiming for “responsive regulation”. Assessing the impact of regulatory actions and adjusting these to actual records of (non) compliance prevents the emergence of a vicious cycle, and can actually build a virtuous cycle where citizens and businesses ask for guidance and support in how best to cooperate. Indeed, a review of evidence on the impact of regulators on organizational cooperation with legal guidelines revealed that regulatory actions were most effective when these enhanced psychological capability, social opportunity and reflective motivation. Accordingly, business inspections generally induced more rule compliance and ethical behaviour than deterrent regulation (Ishwardat et al., 2024).

## Conclusion

We have sketched a multi-level analytical framework that improves our understanding of the understudied problem of cooperation sustainability in families, communities, and organizations – the meso-level realm of society. This framework’s theoretical key contribution consists in explicitly identifying sustainable cooperation as cooperation that is stable as well as valuable and in identifying three distinct threats to such cooperation. Whereas these threats are usually studied in isolation, we explicate the individual and institutional dynamics that affect the stability and value of cooperation. In particular, we show how the framework explains whether or not cooperation at the meso-level will break down or persist.

Individual dynamics (due to contextual shifts in social identities, networks and goals; Spears, 2021) and institutional dynamics that prevent necessary reforms (Van Bavel et al., 2018) impact on the sustainability of meso-level arrangements to support cooperation in families, organizations or communities. Our conceptualization of cooperation sustainability clarifies that threats to sustainability emerge when the stability of the cooperation, the value of the cooperation or both are challenged by external shocks, negative spillover or vicious cycles. Our analysis offers insights on the social origins of individual level concerns, needs, and preferences that help predict which sustainability threats are likely to arise, when these challenge existing social arrangements at the meso-level, and which institutional adjustments need to be made to sustain cooperation for the benefit of society.

Systematically monitoring such threats and exploring possibilities for institutional adaptation at the meso-level, will create opportunities for “new” forms of governance in organizational or community settings (Kolbjørnsrud, 2018; Hirst, 2013) – that are better suited to sustain cooperation by offering continuous adaptability to changing values and social realities.

## Data Availability

Not applicable.
